# Thermographic Analysis of the Metacarpal and Metatarsal Areas in Jumping Sport Horses and Leisure Horses in Response to Warm-Up Duration

**DOI:** 10.3390/ani11072022

**Published:** 2021-07-06

**Authors:** Iwona Janczarek, Witold Kędzierski, Ewelina Tkaczyk, Beata Kaczmarek, Jarosław Łuszczyński, Karolina Mucha

**Affiliations:** 1 Department of Horse Breeding and Use, Faculty of Animal Sciences and Bioeconomy, University of Life Sciences in Lublin, ul. Akademicka 13, 20-950 Lublin, Poland; iwona.janczarek@up.lublin.pl (I.J.); ewelina.tkaczyk@up.lublin.pl (E.T.); khiuk@up.lublin.pl (K.M.); 2 Department of Biochemistry, Faculty of Veterinary Medicine, University of Life Sciences in Lublin, ul. Akademicka 12, 20-033 Lublin, Poland; 3 Department and Clinic of Animal Internal Diseases, Faculty of Veterinary Medicine, University of Life Sciences in Lublin, ul. Akademicka 13, 20-950 Lublin, Poland; beatakaczmar1@gmail.com; 4 Department of Genetics, Animal Breeding and Ethology, Agricultural University, Al. Mickiewicza 24/28, 30-059 Cracow, Poland; jaroslaw.luszczynski@urk.edu.pl

**Keywords:** body surface temperature, exercise, limb temperature, rectal temperature

## Abstract

**Simple Summary:**

A warm-up prepares the body for effort by improving blood supply to muscles and increasing the flexibility of joints, ligaments, and tendons. Warm-up is considered as a necessary preliminary step of each training session. This study assessed the impact of different regimes of warm-up on the surface temperature of the distal parts of limbs in horses used for jumping and leisure riding. Six showjumping horses and six leisure horses were included in the study. The studied horses were warmed up by walking and trotting for various time periods. The rectal temperature and body surface temperature of the distal parts of the four limbs were measured before warm-up, just after it, and during recovery. The warm-up-induced increase in analysed temperatures was higher in jumping sport horses than in leisure horses.

**Abstract:**

This study aimed to assess the impact of various types of warm-up on the metacarpal and metatarsal surface temperature in jumping sport horses in comparison to leisure horses, which work usually less intensively. Six clinically healthy sport geldings, contestants in showjumping competitions, and six geldings used for leisure riding were included in the study. The experiment was conducted for four consecutive days, during which the horses were warmed up by walking and trotting for various durations. Images were taken with a FLUKE Ti9 thermal imager to determine the resting, post-effort, and recovery temperature of the dorsal and plantar surface of the metacarpus and metatarsus of the four limbs. The obtained data were analysed with SmartView 4.1. software. The increase of measured rectal and surface temperatures was proportional to the warm-up duration. The surface temperature increase in the distal limb parts in jumping sport horses was greater than in horses used for leisure. The plantar surface was also warmer than the dorsal surface of the metacarpal/metatarsal areas, with a forelimb being warmer than a hind limb. Elevated temperatures after warm-up persist for 30 min in the recovery period, especially in jumping sport horses compared to leisure horses. Thus, the warming up effect is achieved earlier and lasts longer in heavily trained horses than in non-performance horses.

## 1. Introduction

A warm-up is a necessary part of each training session. It aims to prepare the body for effort by increasing the heart rate, improving blood supply to muscles, and increasing the flexibility of joints, ligaments, and tendons [1]. A warm-up not only improves body performance, but it also prevents injuries due to increased elasticity of the tendon tissues [2]. Injuries usually occur in horses in tendons of the forelimb distal parts [3]. Forelimbs carry about 60% of the horse’s body weight and their load increases during cantering and jumps [4]. Injuries to hind limb tendons are reported less frequently, but their treatment and rehabilitation are as challenging as with the forelimbs [5,6,7,8].

According to Thorpe et al. [4], most injuries originate in the superficial digital flexor tendon when it is extremely stressed. Other factors, such as hyperthermia of the tendon and low oxygen pressure in this tissue, could also lead to tendon damages [9]. In particular, repetitive exposure to hyperthermia decreased a survival rate in tenocytes and induced metabolic disturbances in surviving cells [9,10,11]. Although the probability of cell death caused by high temperatures in vivo is low, their frequent exposure to overheating impairs cell metabolism and initiates degenerative processes in the tendon structure, subsequently predisposing it to mechanical failure [9]. On the other hand, the use of protection boots or bandages on limbs results in an exercise-induced increase in surface temperature of up to 17 °C for distal limb parts [12]. Nevertheless, a tendon warm-up applied for the optimum intensity and duration improves its elasticity [13]. 

Although the use of warm-up in equestrianism is beyond dispute, there have been few studies into its effect on horse performance. According to Stachurska et al. [14], the warm-up duration had no impact on the final result of the jumping competition while older showjumping horses obtained a shorter warm-up than younger horses. Murray et al. [15] reported that warm-up duration increased with the level of dressage competition and can be positively associated with final score. Moreover, differences in the warm-up pattern depend, among others, on the riding discipline which the horse was trained for [16]. So far, there is very little published data on standards for the use of warm-up in athletic horses [17]. 

It is known that in response to exercise, the muscles produce additional amounts of heat due to increased metabolism and mechanical work. The heat is spread to the skin by the circulating blood and conduction, causing an increase in the internal and body surface temperature in proportion to the intensity of exercise [18]. The body surface temperature could be monitored using infrared thermography, a noninvasive imaging method [18]. It can be used to evaluate changes in body surface temperatures associated with the circulatory system, physiological system, or arising due to inflammatory processes [19]. Therefore, this method is used in veterinary practice to detect tendon injuries [9,18]. However, the results obtained from thermography are influenced by many internal and external conditions, such as horse breed and training level, time of day, ambient temperature, insolation, airflow, etc.; thus, this method still needs to be refined in order to be applicable in monitoring the course of horse training [20,21]. Moreover, the number of publications reporting dynamic changes in the body surface temperature in healthy horses is limited; therefore, it is not possible to compare and analyse data of this kind [22]. Furthermore, although the warm-up is used before each training session and competition, no research-based guidelines have yet been developed regarding its protocol depending on the horse performance. In particular, limb surface temperature has not been studied in this aspect.

The hypothesis was posed in the study that a minimum warm-up time resulting in a significant rise in the temperature of studied horses’ legs areas depends on the horse’s performance. Therefore, this study aimed to assess the impact of warm-up of various durations on the metacarpal and metatarsal surface temperature and the rectal temperature in jumping sport horses—used as an example of heavily trained, athletic horses—in comparison to non-performance, leisure horses, which work usually less intensively. It can be assumed that the obtained results will be useful in practice in adapting the duration of the warm-up to the manner in which the horses are used. Data obtained in this study would be used as a basis for future thermographic research to develop standards for the use of warm-up in athletic horses.

## 2. Materials and Methods

### 2.1. Horses

Six geldings used for leisure riding only and six geldings used for showjumping sport were included in the study. The studied horses were warmblood, 6–8 years old, and weighed 553 ± 60.5 kg. All horses had been used for riding for at least 24 months. The sport horses had been trained in jumping for at least 12 months and competed in national competitions in showjumping at a height of 120 cm. The horses included in the study did not have a history of musculoskeletal injuries in the distal limb. The study was conducted in an equestrian center used for scientific research, where the horses were under constant veterinary care and the history of their training and veterinary treatments was well documented. The pattern of everyday work (before the experiment period), each time lasting about 60 min, is shown in Table 1.

All horses were housed individually in boxes (3.5 × 3.5 m) divided with open work walls. There was a manger and an automatic water bowl in each box. The floor was bedded with wheat straw every day. The horses were fed three times daily with hay and rolled oats supplemented with minerals and vitamins. Fodder rations were adjusted individually for each horse, depending on the work intensity. Sport horses were administered complete granulated feed instead of oats at noon. Each horse had been examined by a veterinarian one day before the experiment, and was found to not show any lameness or other health-disturbing symptoms.

### 2.2. Experiment

The experiment was conducted on a 30 × 50 m outdoor arena with a professional riding surface (sand with the addition of fibres). The arena was the standard place of everyday exercise for the experimental horses. The experiment was carried out for four consecutive days, during which the horses were warmed up by walking and trotting with various durations (Table 2). Both at walk and in trot, the horses changed the direction of work every 2.5 min. The work was carried out at the same time each day. The weather conditions were stable: cloudless, air temperature: 20 °C (±1.6), relative humidity: 43% (±2.1), wind velocity: 0.4 m/s (±0.1), atmospheric pressure: 1011 hPa (±3.4). The riders taking part in the experiment were of the same gender: they were two horsewomen equally advanced in their riding skills (they could take part in 120 cm jumping competitions) and their average body weight was 62 kg (± 3.7 kg). During the study, none of the horses was shod and horses were exercised without boots or bandages on the limbs. During the 4-day period of the study, apart from the tested warm-up, the horses spent three hours a day on a paddock and the rest of the time in their stall.

### 2.3. Measurement Times 

Measurements of rectal and body surface temperature were taken at rest, i.e., 15 min before each warm-up (resting temperature), immediately after the end of warm-up (post-effort temperature), and 30 min later (recovery temperature). Between the post-effort and last measurement, the horses were led at walk by hand, which lasted about 25 min. After the warm-up and the post-effort measurements, the horses were walked to their boxes in the stable. 

### 2.4. Measurements of Inner Body Temperature

The inner body temperature of the horses was measured rectally with a veterinary thermometer (Veterinär–Thermometer SC 12, Langeskov, Denmark). The measurement duration was 30 s, as recommended by the thermometer manufacturer. Measurements were carried out inside an indoor arena simultaneously with the thermographic measurement.

### 2.5. Thermographic Measurements

**Figure 1:**
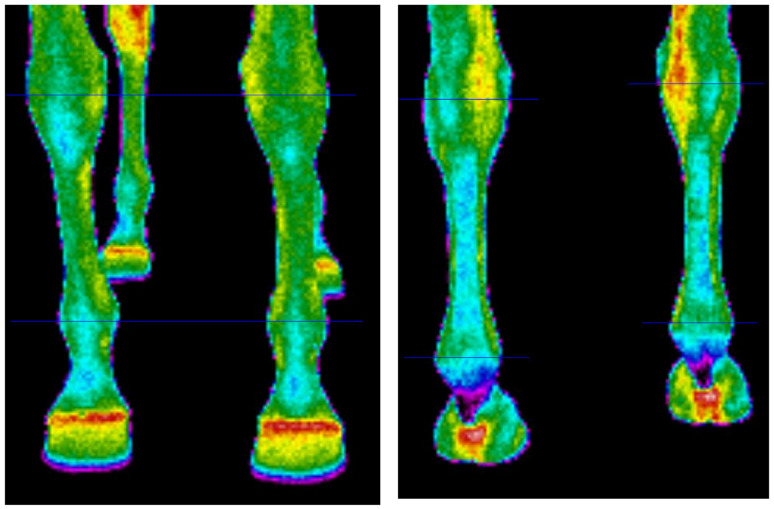
Example of thermographic images of forelimbs in the dorsal (**left panel**) and plantar (**right panel**) views, illustrating thermographic investigations performed. Regions of interest (ROIs) are between horizontal lines.

The thermographic method was applied to determine the surface temperature of the metacarpus and metatarsus areas in the four limbs. The images were taken with a FLUKE Ti9 thermal imager, with an uncooled microbolometer sensor and a focal plane array of 120/160 pixels, with an infrared spectral band of 7.5–14 μm (Fluke Corporation, Everett, Washington, DC, USA) [23]. The standard procedures were followed during the image taking, i.e., the horse was in a dark and windless place, with a relatively constant air temperature [18,24]. The measurements were taken in an indoor arena, which was well known to horses, situated between the outdoor arena and the stable. The indoor arena was closed and shadowed to prevent heat inflow from the outside such as solar radiation or airflow. It was draught-free with stabilized temperature of 19 °C and relative air humidity of 42–45%. The camera was situated 120 cm away from the horse’s body, 15 cm above the ground, at a 90° angle to the coronal plane of each limb individually. Thus, IFOV of used camera at 120 cm amounted to 3 mm. Images of the dorsal and the plantar part were taken for each limb. After the images were taken, the camera data were uploaded to the computer and analysed in SmartView 4.1 software. On each obtained thermographic image, the region of interest (ROI) was defined. It was the limb area bounded by horizontal lines through the midpoints of the knee and fetlock joint of the dorsal and the plantar part of fore limbs, and hock and fetlock joint of the dorsal and the plantar part of the hind limbs (Figure 1). The obtained value of surface temperature was the average temperature of the analysed ROI.

### 2.6. Statistical Methods

All of the statistical analyses were conducted with STATISTICA v. 10 (StatSoft, Tulsa, OK, USA). Data distribution does not differ significantly from the theoretically normal distribution according to the Shapiro-Wilk test at *p* < 0.05. Images of the right and left limbs were obtained for each horse, then the homologous contralateral regions were compared using the paired Student’s test. Due to the lack of significant differences, mean values of measurements of these two sides of the body were taken for future analysis. Therefore, the statistical analysis was based on multivariate analysis of variance (ANOVA GLM), taking into account the warm-up duration (*n* = 4: very short, short, extended, long-lasting), time of measurement (resting, post-effort, recovery), the rider effect, method of horse use (*n* = 2: leisure, jumping sport), limb part (*n* = 2 distal part of the forelimb, distal part of the hind limb), measurement area (*n* = 2: dorsal, plantar), and interactions between the factors. The rider effect was statistically insignificant. The significance of differences between the mean values was determined by Tukey’s test (*p* ≤ 0.05). Due to the small number of horses used in the study, the power analysis of the test was performed. Assuring the test power at the level of 0.80, the number of data was found to be enough to achieve the adopted significance level (α) as lower than 0.05.

## 3. Results

There were no statistically significant differences between the rectal temperature in leisure and sport horses in measurements before warm-ups of different duration (Table 3). The mean temperature was 37.61 ± 0.21 °C. Each type of warm-up was followed by a significant increase in the rectal temperature. This temperature was significantly higher after a long-lasting warm-up in leisure horses than after the other warm-up types (Table 3). The analysed parameter was significantly lower in the jumping sport horses following a very short warm-up than after other warm-up types. There were no significant differences noted between horses used in different ways only following a very short warm-up. The rectal post-effort and recovery temperatures of jumping sport horses in the other cases were significantly higher than for horses used for leisure. In leisure horses, the recovery rectal temperature did not differ significantly from the resting temperature. In jumping sport horses, in all types of warm-up studied, the recovery rectal temperatures were lower than the post-effort rectal temperature, although they were higher than resting values.

The resting temperatures of the metacarpal and the metatarsal dorsal surfaces and metatarsal plantar surface in jumping sport horses were significantly higher than those limb surfaces in leisure horses (Table 4). Moreover, post-effort and recovery temperatures of the dorsal and plantar areas of metacarpus in the leisure horses were significantly higher than those of metatarsus. There were cases in which significantly higher post-effort and recovery temperatures were noted in some areas in sport horses compared to the corresponding areas in leisure horses. Therefore, the effect of the warm-up duration under study was considered separately for each of the studied surface areas.

No significant differences in the resting temperature of the analysed individual surfaces (e.g., the dorsal surface of the metatarsus) were observed in measurements performed before warm-ups of different duration (Table 5). Each type of warm-up resulted in a surface temperature increase in leisure horses. It was similar in jumping sport horses, except in the dorsal area of the metacarpus following a very short and short warm-up and in the dorsal area of the metatarsus following a very short warm-up. The post-effort temperature following a long-lasting warm-up in leisure horses was usually higher than the temperature recorded after shorter warm-up regimes. The plantar area of the metacarpus, where an extended warm-up resulted in significantly higher temperatures than shorter warm-up types, was an exception. The type of warm-up in jumping sport horses had a greater impact on the temperature of the analysed limb areas. In general, longer-lasting exercises resulted in significantly greater temperature increases. A 30-min recovery period proved insufficient for the post-effort temperature increase to return to the resting level. The dorsal area of the metatarsus in jumping sport horses after a very short warm-up was an exception. Recovery temperatures in leisure horses following a long-lasting warm-up, and those of the plantar area of the metacarpus following an extended warm-up, were significantly higher than after shorter types of exercise. In jumping sport horses, the recovery temperatures of metacarpus dorsal and plantar areas obtained after extended and long-lasting warm-up were significantly higher than after shorter warm-up types and the temperatures of metatarsus dorsal and plantar areas obtained after short and extended warm-up were higher than after very short warm-up, whereas the recovery temperatures of the discussed areas after long-lasting warm-up were higher than after others warm-up types. The surface temperature of the analysed limb areas in leisure horses was significantly lower in most cases than in jumping sport horses.

## 4. Discussion

The resting rectal temperature of the analysed horses remained in the reference range (37.5 to 38.5 °C) [21,25,26]. This parameter did not differ for various studied warm-up types or types of horse use and can be considered as the baseline rectal temperature. The differences in the rectal temperature related to the warm-up type were recorded after exercise. It is particularly noteworthy that the post-warm-up values of internal body temperature were higher in jumping sport horses than in leisure horses. The rectal temperature in leisure horses returned to the resting level after a 30-min recovery period, except after a long-lasting warm-up. The recovery temperatures in jumping sport horses were already elevated after a light warm-up. The observed differences in post-exercise temperature values in the studied groups of horses can be the result of differences in the metabolism intensity and also a larger stroke volume of the heart in well-trained subjects [27].

A comparison of the resting surface temperature of the analysed ROIs showed no significant differences on individual experiment days. These findings are not surprising because the resting surface temperature in clinically healthy horses is known to be constant while it is measured in constant environment conditions [28]. Generally, the resting surface body temperature of a horse may be affected by such factors as the quality of the ground in the stall, microclimate in the stable, weather conditions or the length and thickness of the hair cover [29,30]. However, the external conditions were stable during the study, and thus they did not influence the obtained results. Therefore, the results obtained at rest can be considered as the baseline temperature.

A lower resting temperature in distal limb parts in leisure horses as compared to such temperature in jumping sport horses proved to be consistent with the findings of a study conducted by Soroko et al. [20], who demonstrated the significant impact of a horse’s training level on the temperature of distal parts of limbs and some areas of the back, which were higher in well-trained horses than in horses in the initial phase of training. Similarly, it was found in Thoroughbred horses that the surface temperature of metacarpus and metatarsus areas determined at rest increased with the intensity of exercise used during the training season [31]. The studied jumping sport horses were routinely submitted to more demanding exercises than leisure horses, according to their type of use [32,33]. Physiologically, the body surface emits heat generated continuously through the deeper tissues and this heat spreads to the skin by the conduction and circulating blood [34]. Soft tissues produce heat as a side effect of metabolic changes. As Turner and Pansch [35] suppose, horses undergoing training, especially jumping horses, are placed under great strain, which can lead to microinjuries in the musculoskeletal system, including tendons in distal parts of the limbs. Local inflammation and the processes of tissue reconstruction and repair could induce accelerated metabolism and increased blood supply, resulting in the release of greater amounts of heat. Nevertheless, the studied horses did not show any health-disturbing symptoms.

The studied types of warm-up resulted in a temperature increase in the analysed limb surfaces proportionally to its duration. An increase in the plantar limb surface temperatures following effort was also observed by other researchers [12,36]. The temperature of the skin in the metacarpus plantar and the metatarsus plantar area increased by approx. 3 °C after 22.5 min of exercise, including 5 min of trotting and 2.5 min of cantering [36]. No impact was observed for exercise consisting of 5 min of walking, 5 min of trotting, 5 min of walking, and 5 min of trotting [12]. Therefore, the exercise protocol has an immediate effect on the temperature increase in the studied body areas. Thus, the findings of this study can be regarded as consistent with those cited. The longer the exercise time, the higher increase in the temperature of the analysed limb areas.

Moreover, it was found that the post-effort surface temperature in leisure horses was significantly higher after a long-lasting warm-up than after other, shorter types of exercise, while in sport horses, significantly higher temperatures of the majority of analysed areas were observed just after a short warm-up, compared to very short. Therefore, from the point of view of thermographic analysis of dorsal parts of limbs, it seems that a short warm-up in sport horses lasting 25 min was enough to achieve its effect. Thus, probably the warm-up period in jumping sport horses can be limited to 25 min, including 10 min of trotting. The warm-up duration practiced for elite showjumping horses reported in another study amounted to 12 to 27 min including cantering [37]. However, a temperature increase in the dorsal area of the metacarpus cannot be expected until after an extended warm-up. Hence, this type of warm-up was regarded as sufficient to warm up the whole area of distal limb parts, even the dorsal part of the metacarpus as the area most resistant to warming up. There is a lack of similar thermographic studies performed on horses with which the results obtained in the present study could be compared.

A warm-up resulted in greater increases in studied internal and body surface temperatures in jumping sport horses than in leisure horses. It seems that the findings can be explained by the body becoming accustomed to a specific type of effort due to a specific training [38,39]. For leisure horses, the effort level is submaximal sporadically and for a short time. Usually, it is monotonous work of light or moderate intensity [40]. The importance of this issue was also emphasised by Webbon [41], who demonstrated considerable tendon injuries in 26.1% of horses examined postmortem. An increase in the surface temperature in the tendon area compared to the rest of the metacarpus and metatarsus area can be regarded as beneficial as this improves the tendon elasticity and joint mobility [42]. However, prolonged and frequent increase in the distal limb part temperature can result in tendon injury [10,43]. Moreover, it can be supposed that increased temperature of the hind limbs in sport horses just after a short warm-up is caused by their intensive work, which is necessary in jumping horses when they approach an obstacle and leap. Increased load on the limbs by showjumping horses becomes natural during movements [44]. The plantar part of distal limbs is the most loaded and stressed during the landing after a jump [45]. Thus, an increased metabolism and heat production in loaded parts of legs are probably the reason for increased surface temperatures of these areas in sport-jumping horses after low-intensity warm-up as compared to leisure horses.

The higher surface temperature of distal parts of the forelimb compared to the hind limb, especially noticeable in the dorsal areas of leisure horses, was also stated. This is probably associated with the natural position of the centre of gravity of their bodies being closer to the forelimbs which causes the forelimbs to sustain about 60% of the body mass of the sound horse [46]. The differences are noticeable in leisure horses, which are not required to be collected when working under a rider [47]. This adverse position of the centre of gravity can be adjusted when self-carriage and balance is achieved, which is practised in advanced training [48].

A lower post-effort temperature of both pairs of limbs in sport and leisure horses was observed in the dorsal area compared to the plantar area. This may have been caused by a larger load on flexor tendons compared to the extensor tendons, which is especially visible in the more loaded forelimb. The digital flexor tendons have a larger cross-section than the corresponding extensor tendons. Therefore, the tendon area with poor blood supply contributes to a lower temperature of the plantar parts compared to the dorsal parts while measured at rest. After the exercise, since part of the mechanical energy transmitted through the tendons is transformed into heat, higher temperatures are observed on the body surfaces covering the flexor tendons. These findings can be regarded as consistent with those presented by Soroko et al. [20], who examined young Thoroughbred racehorses. Therefore, it seems that the relationship is typical of the species and is not associated with the type of horse use.

The results also indicated that the surface temperatures were elevated during the recovery period. The recovery temperatures in jumping sport horses were already elevated after a low-intensity warm-up and not until after a long-lasting warm-up in leisure horses. This situation not only reflects post-effort results, but it also shows that cooling down the distal parts of horse limbs after exercise to lower their temperature is reasonable [49]. However, it appears that cooling down is primarily needed for horses in sports training, regardless of the type of exercise applied during training. Such treatments on leisure horses, on the other hand, should be performed when the effort they exerted was much more exacting than the daily practice. According to Thorpe et al. [4], a long-term temperature increase in the tendon area can provoke its injury. Therefore, cooling down in these cases seems to be necessary.

Some limitations of the study include the fact that the used work-load in all warm-up regimes was lower than generally used in practice in showjumping and dressage horses [37,50]. The reported warm-up practices included intervals of cantering. Although the low-intensity warm-ups described in other research studies included 10 min of trotting (similar to the present study), a warm-up of higher intensity included cantering [51,52]. Thus, further studies with the use of canter as an element of warm-up should be conducted. Nevertheless, the used warm-up regimes were sufficient to induce a significant increase in analysed internal and body surface temperatures. Another limitation of the study could be the relatively small number of horses studied; however, this number was sufficient to find significant differences in the response to the studied types of warm-up in jumping and leisure horses.

## 5. Conclusions

The surface temperature in the distal limb parts in jumping sport horses is higher than in leisure horses regardless of the warm-up regime. The plantar part is warmer in response to warm-up than the dorsal part of the metacarpus/metatarsus, with the forelimbs being warmer than the hind limbs, especially in leisure horses, while in jumping sport horses these differences are less pronounced. The very short and short warm-up (lasting 25 min, including 10 min of trotting) was insufficient to significantly increase the temperature of metacarpus dorsal area. In sport horses, the significantly highest temperatures of all studied areas of the limbs were reached after 30 min of warm-up, while in leisure horses a similar effect appeared only after 35 min of warm-up. On the other hand, during 30-min recovery after a 20-to-30-min warm-up, the internal body temperature dropped to its resting value in leisure horses, while it remained at an elevated level in the sport horses studied. Thus, the warming-up effect is achieved earlier and lasts longer in heavily trained horses than in non-performance horses.

## Figures and Tables

**Table 1:** Weekly cycle of horses’ work.

Day of the Week	Leisure Horses	Jumping Sport Horses	
		Week of Routine Training	Week with Competition
Monday	Paddock	Dressage ^3^	Walk ^5^
Tuesday	Walk, trot, canter ^1^	Jumping ^4^	Jumping ^4^
Wednesday	Walk, trot, canter ^1^	Dressage ^3^	Dressage ^3^
Thursday	Walk, trot, canter ^1^	Jumping ^4^	Jumping ^4^
Friday	Walk, lunging ^2^	Dressage ^3^	Walk ^5^
Saturday	Walk, trot, canter ^1^	Jumping ^4^ or cross-country riding ^1^	Showjumping competition
Sunday	Cross-country riding ^1^ (excluding jumping)	Paddock	Showjumping competition or walk ^5^

^1^—mean speed about 2.5 m/s; ^2^—mean speed: 1.2 m/s; ^3^—technical training of some dressage tasks such as responsiveness and collection in walk, trot, and canter with mean speed of 3.2 m/s; ^4^—warm-up, 30 to 40 jumps, maximum height of the obstacles: 1.2 m, mean speed during jumping course: 4.6 m/s; ^5^—mean speed: 1.6 m/s.

**Table 2:** Warm-up regime (min) on consecutive experiment days.

Day of Experiment	Walk 1.6 m/s	Trot 3.2 m/s	Walk 1.6 m/s	Total Duration of Warm-Up	Warm-Up Regime (Author’s Nomenclature)
1.	10	5	5	20	Very short warm-up
2.	10	10	5	25	Short warm-up
3.	10	15	5	30	Extended warm-up
4.	10	20	5	35	Long-lasting warm-up

**Table 3:** Horse rectal temperature (°C) depending on the type of warm-up (mean ± SD).

Type of Warm-Up	Leisure Horses (*n* = 6)			Jumping Sport Horses (*n* = 6)		
	A	B	C	A	B	C
Very short	37.64 ± 0.23 x	38.56 ± 0.34 ya	37.98 ± 0.36 x	37.55 ± 0.22 x	38.78 ± 0.36 ya	38.12 ± 0.38 za
Short	37.59 ± 0.29 x	38.44 ± 0.41 ya	37.97 ± 0.41 x	37.56 ± 0.27 x	39.45 ± 0.29 yb *	38.67 ± 0.40 zb *
Extended	37.65 ± 0.25 x	38.49 ± 0.38 ya	37.92 ± 0.37 x	37.56 ± 0.25 x	39.52 ± 0.32 yb *	38.77 ± 0.41 zb *
Long-lasting	37.71 ± 0.25 x	39.12 ± 0.33 yb	38.17 ± 0.32 z	37.62 ± 0.23 x	39.66 ± 0.37 yb *	38.69 ± 0.36 zb *

A—values obtained at rest, B—immediately after the end of warm-up, C—30 min after the end of warm-up. Means marked with different letters differ significantly at *p* < 0.05: x,y,z—in rows, between A, B, and C; a,b,c—in columns, between the types of warm-up; *—value higher in jumping sport horses in comparison to leisure horses.

**Table 4:** Resting temperature (°C) of the limb surface area under analysis of all warm-up regimes (mean ± SD).

Analysed Surface Area	Leisure Horses (*n*= 24)			Jumping Sport Horses (*n* = 24)		
	A	B	C	A	B	C
Metacarpus dorsal	28.51 ± 0.51 a	31.72 ± 1.21 a	31.27 ± 0.97 a	30.42 ± 0.53 a *	31.82 ± 1.35 ab	31.44 ± 1.19 ab
Metatarsus dorsal	27.71 ± 0.46 b	29.36 ± 0.79 b	29.02 ± 0.93 b	30.07 ± 0.51 a *	31.44 ± 1.01 a *	30.44 ± 1.26 a *
Metacarpus plantar	28.10 ± 0.49 ab	33.12 ± 1.42 c	32.53 ± 1.40 a	28.72 ± 0.48 b	33.50 ± 1.33 b	32.60 ± 1.03 b
Metatarsus plantar	28.49 ± 0.50 a	30.95 ± 1.48 ab	30.15 ± 1.57 b	29.17 ± 0.49 b *	32.55 ± 1.47 ab *	31.25 ± 1.64 ab

*n*—number of measurements (number of horses multiplied by four days of the study), A—values obtained at rest, B—immediately after the end of warm-up, C—30 min after the end of warm-up. Means marked with different letters differ significantly at *p* < 0.05: a,b,c—in columns, between analysed surfaces; *—value higher in jumping sport horses in comparison to leisure horses.

**Table 5:** Temperature (°C) of the analysed limb surface area depending on the warm-up regime (mean ± SD).

Type of Warm-Up	Leisure Horses			Jumping Sport Horses		
A	B	C	A	B	C
Metacarpus dorsal area						
Very short	28.54 ± 0.61 x	31.12 ± 0.66 ya	30.83 ± 0.54 ya	30.33 ± 0.55 *	30.78 ± 0.58 a	30.55 ± 0.49 a
Short	28.42 ± 0.50 x	31.22 ± 0.48 ya	31.01 ± 0.51 ya	30.64 ± 0.69 *	30.77 ± 0.67 a	30.63 ± 0.61 a
Extended	28.53 ± 0.48 x	31.45 ± 0.46 ya	31.07 ± 0.44 ya	30.71 ± 0.75 x *	32.76 ± 0.69 yb *	32.24 ± 0.73 yb *
Long-lasting	28.55 ± 0.71 x	33.08 ± 0.54 yb	32.17 ± 0.57 zb	29.98 ± 0.79 x *	32.98 ± 0.65 yb	32.33 ± 0.69 zb
Metatarsus dorsal area						
Very short	27.82 ± 0.54 x	29.07 ± 0.65 ya	28.77 ± 0.63 ya	30.17 ± 0.45 x *	30.59 ± 0.71 xa *	29.39 ± 0.54 ya *
Short	27.67 ± 0.67 x	29.11 ± 0.57 ya	28.63 ± 0.52 ya	29.99 ± 0.56 x *	31.33 ± 0.67 yb *	30.12 ± 0.64 xb *
Extended	27.88 ± 0.52 x	29.36 ± 0.74 yab	28.86 ± 0.64 ya	29.87 ± 0.34 x *	31.67 ± 0.63 yb *	30.50 ± 0.61 zb *
Long-lasting	27.45 ± 0.56 x	29.89 ± 0.66 yb	29.83 ± 0.55 yb	30.23 ± 0.61 x *	32.17 ± 0.70 yb *	31.73 ± 0.58 yc *
Metacarpus plantar area						
Very short	28.04 ± 0.37 x	32.02 ± 0.78 ya	31.41 ± 0.74 ya	28.47 ± 0.57 x	32.02 ± 0.60 ya	31.76 ± 0.61 ya
Short	28.22 ± 0.57 x	32.16 ± 0.72 ya	31.63 ± 0.55 ya	29.05 ± 0.47 x *	33.16 ± 0.63 yb *	32.36 ± 0.63 zb *
Extended	28.24 ± 0.44 x	34.08 ± 0.62 yb	33.43 ± 0.67 yb	28.66 ± 0.70 x	34.03 ± 0.72 ybc	33.03 ± 0.52 zc
Long-lasting	27.87 ± 0.61 x	34.22 ± 0.67 yb	33.63 ± 0.73 yb	28.69 ± 0.58 x *	34.77 ± 0.69 yc	33.23 ± 0.63 zc
Metatarsus plantar area						
Very short	28.63 ± 0.56 x	30.13 ± 0.54 ya	29.49 ± 0.57 ya	29.18 ± 0.45 x	30.41 ± 0.66 ya	29.07 ± 0.61 xa
Short	28.46 ± 0.49 x	30.02 ± 0.65 ya	29.16 ± 0.59 za	29.18 ± 0.44 x *	32.55± 0.71 yb *	31.34 ± 0.65 zb *
Extended	28.54 ±0.62 x	30.17 ± 0.72 ya	29.41 ± 0.65 za	29.22 ± 0.34 x *	33.45 ± 0.77 yc *	31.67 ± 0.72 zb *
Long-lasting	28.31 ± 0.54 x	33.49 ± 0.69 yb	32.55 ± 0.64 zb	29.11 ± 0.58 x *	33.78 ± 0.68 yc	32.92 ± 0.61 zc

A—values obtained at rest, B—immediately after the end of warm-up, C—30 min after the end of warm-up. Means marked with different letters differ significantly at *p* < 0.05: x,y,z—in rows, between A, B, and C; a,b,c—in columns, between the types of warm-up; *—value higher in jumping sport horses in comparison to leisure horses.

## Data Availability

Data sharing is not applicable to this article.
